# Therapeutic potential of spexin in diabetic neuropathy: Insights from in vitro and in vivo models

**DOI:** 10.1113/EP093879

**Published:** 2026-06-08

**Authors:** Mehmet Refik Bahar, Seval Ulku Orhan, Cigdem Tekin, Asli Taslidere, Mete Ozcan, Suat Tekin

**Affiliations:** ^1^ Department of Nutrition and Dietetic, Faculty of Health Science Osmaniye Korkut Ata University Osmaniye Turkey; ^2^ Department of Biophysics, Faculty of Medicine Izmir Bakırcay University Izmir Turkey; ^3^ Department of Home Patient Care, School of Health Services Inonu University Malatya Turkey; ^4^ Department of Histology and Embryology, Faculty of Medicine Inonu University Malatya Turkey; ^5^ Department of Biophysics, Faculty of Medicine Firat University Elazig Turkey; ^6^ Department of Physiology, Faculty of Medicine Inonu University Malatya Turkey

**Keywords:** diabetes, diabetic neuropathy, inflammation, oxidative stress, spexin

## Abstract

The present study investigated the therapeutic potential of spexin (SPX) on both in vitro and in vivo models of diabetic neuropathy (DN). A total of 40 male BALB/c mice were randomly allocated into four experimental groups (*n* = 10 per group): a normoglycaemic control, an untreated DN group and two SPX treatment groups (DN+SPX‐12.5 and DN+SPX‐25). To induce the diabetic state, subjects received a single intraperitoneal (i.p.) injection of streptozotocin (STZ) at a dosage of 150 mg/kg. Following the establishment of DN, the treatment groups were administered SPX (12.5 or 25 µg/kg/day) via i.p. injection for 15 consecutive days, whereas the untreated DN group received an equivalent volume of saline vehicle. Thermal hyperalgesia and mechanical allodynia were subsequently evaluated utilizing the hot plate, tail‐flick and electronic von Frey behavioural paradigms. in vitro assays demonstrated that SPX administration significantly restored the viability of high glucose‐exposed dorsal root ganglion cells, while in vivo assessments revealed a marked attenuation of mechanical allodynia and thermal hyperalgesia in SPX‐treated diabetic mice. Furthermore, SPX exhibited profound antioxidant and immunomodulatory properties by mitigating systemic oxidative stress and pro‐inflammatory cytokine responses, concomitantly preserving the structural integrity of the pancreatic islets. Taken together, these findings provide compelling evidence that SPX confers neuroprotective benefits in STZ‐induced DN through the modulation of oxidative stress, neuroinflammation and nociceptive sensitization, thereby highlighting its potential as a novel therapeutic agent for diabetic microvascular complications.

## Abstract (highlights)

- **What is the central question of this study?**
  What is the therapeutic, neuroprotective and antinociceptive potential of the neuropeptide spexin (SPX) in in vitro and in vivo models of diabetic neuropathy?
- **What is the main finding and its importance?**
  SPX effectively alleviates thermal hyperalgesia and mechanical allodynia while directly preserving the viability of high‐glucose‐exposed dorsal root ganglion neurons. Additionally, it significantly reduces systemic oxidative stress and neuroinflammation, and protects pancreatic β‐cells. These results highlight SPX as a promising novel therapeutic candidate for managing painful diabetic microvascular complications.

## 1 INTRODUCTION

Diabetes mellitus (DM) represents a heterogeneous cluster of metabolic disorders characterized by chronic hyperglycaemia resulting from defects in insulin secretion, resistance to insulin action or a combination of both (Savelieff et al., 2025). Far beyond a simple disruption of glucose homeostasis, the chronic hyperglycaemic state induces a systemic metabolic dysregulation that profoundly affects carbohydrate, lipid and protein metabolism. This persistent elevation of systemic glucose levels destabilizes homeostatic balance by triggering oxidative stress and disrupting cellular signalling pathways (Eftekharpour & Fernyhough, 2022). Consequently, DM evolves into a progressive and devastating multisystemic pathology, manifesting as microvascular complications – such as retinopathy, nephropathy and neuropathy – as well as macrovascular conditions like accelerated atherosclerosis, which remain the primary determinants of morbidity and mortality in these patients (Cole & Florez, 2020). Among these systemic complications, diabetic neuropathy (DN) is recognized as the most prevalent and costly chronic sequela, drastically impairing the quality of life (Tao et al., 2025). Affecting approximately 50% of the diabetic population, DN induces significant structural and functional damage across both the somatic and autonomic components of the peripheral nervous system (Savelieff et al., 2025).

According to the International Diabetes Federation 2025 Atlas, the global diabetic population among adults is projected to rise from 589 million to 853 million by 2050 (Magliano et al., 2025). This alarming trajectory suggests a parallel surge in the prevalence and economic burden of DN. Clinically, DN encompasses a broad spectrum of symptoms ranging from asymptomatic progression to severe neuropathic pain, sensory loss and lower‐limb amputations resulting from diabetic foot ulcers – conditions frequently associated with chronic pain, anxiety, depression and sleep disturbances (Tao et al., 2025).

The pathophysiological foundations of DN‐induced chronic pain and neurodegeneration centre on the vulnerability of the dorsal root ganglion (DRG) neurons (Reinhold & Rittner, 2020). Lacking the full protection of the blood–nerve barrier, DRG neurons are directly exposed to systemic hyperglycaemia and toxic metabolic byproducts (Reinhold & Rittner, 2020). Excessive intracellular glucose entry leads to mitochondrial electron leakage, fuelling the overproduction of reactive oxygen species (ROS), particularly superoxide anions (Eftekharpour & Fernyhough, 2022). This oxidative surge triggers pathological mitochondrial fission, leading to a ‘bioenergetic crisis’ where neurons can no longer sustain axonal transport or energy demands. This cascade ultimately results in neuronal apoptosis and the characteristic ‘dying‐back’ neuropathy observed in distal axons (Eftekharpour & Fernyhough, 2022).

Parallel to neuronal loss, DN is marked by neuronal hyperexcitability and nociceptive sensitization driven by neuroinflammation (Zhu et al., 2023). Metabolic stress activates Schwann cells and satellite glial cells within the DRG, stimulating the release of pro‐inflammatory cytokines such as tumour necrosis factor‐alpha (TNF‐α), interleukin (IL)‐1β and IL‐6 (Zhu et al., 2023). These mediators activate nuclear factor‐κB (NF‐κB) and mitogen‐activated protein kinase (MAPK) pathways, which in turn modulate the activity of nociceptive ion channels (Zhu et al., 2023). The resulting phosphorylation and lowered activation thresholds of these channels lead to ectopic action potentials, manifesting clinically as mechanical allodynia and hyperalgesia (Tao et al., 2025). Furthermore, the progressive loss of pancreatic β‐cells and the subsequent deficiency of C‐peptide – both of which provide critical neurotrophic support and maintain nerve blood flow – further exacerbate this neurodegenerative process (Sima, 2003; Tomlinson et al., 1997).

Spexin (SPX) has emerged as a novel candidate for modulating these complex pathways. Discovered in 2007 (Mirabeau et al., 2007), this highly conserved 14‐amino‐acid neuropeptide is involved in energy metabolism, appetite regulation and antinociception (Lv, Zhou et al., 2019; Sun et al., 2023). SPX exerts its effects primarily through galanin receptor 2 (GalR2) and galanin receptor 3 (GalR3), acting as a potent pain modulator at both central and peripheral levels (Kim et al., 2014). Functional studies have demonstrated that central administration of SPX reduces pain sensitivity in tail‐flick tests and modulates acute inflammatory and visceral pain via the dynorphin/kappa‐opioid receptor pathway. In the context of DM, low serum SPX levels have been clinically correlated with painful DN, while peripheral SPX treatment has shown efficacy in alleviating mechanical hyperalgesia in murine models. Beyond pain modulation, SPX inhibits hypoxia‐induced mitochondrial dysfunction, suppresses pro‐inflammatory cytokines while elevating IL‐10, and enhances pancreatic β‐cell viability (Türkel et al., 2022).

Given the critical need for innovative pharmacological agents that can regulate the oxidative, inflammatory and apoptotic cascades of DN, the present study was designed to investigate the therapeutic potential of SPX. By utilizing both in vitro and in vivo models, we aimed to validate the molecular efficacy of SPX in preserving neuronal survival and mitigating the sensory complications of DN. The fundamental aim of incorporating the in vitro DRG model was to isolate and validate the direct cytoprotective effects of SPX on primary sensory neurons against high glucose‐induced toxicity, independent of its systemic glycaemic or metabolic modulations observed in vivo.

## 2 METHODS

### 2.1 Ethical approval

For the experiments described here, we used a total of 40 adult male BALB/c mice (6–8 weeks old; 30–35 g) and neonatal Wistar Albino rats (postnatal days 1–2) obtained from the Experimental Animal Production and Research Centre of Inonu University (Malatya, Turkey). All experimental procedures were approved by the Local Animal Experimentation Ethics Committee of Inonu University (Decision No: 2024/8‐2). In addition, the principles governing the care and treatment of animals, as stated in the *Guide for the Care and Use of Laboratory Animals* and the ethical frameworks established by the IASP Research and Ethics Committee, were followed at all times during this study (Zimmermann, 1983). Furthermore, this research complies with the animal experiment policies of *Experimental Physiology*. Animals had ad libitum access to tap water and standard rodent chow and were individually housed in a well‐ventilated, temperature‐controlled environment (21 ± 1°C; 60 ± 5% relative humidity; 12 h–12 h light–dark cycle).

### 2.2 in vitro studies

The in vitro phases of the research were performed within the laboratories of the Department of Physiology at Inonu University's Faculty of Medicine and the Department of Biophysics at Firat University's Faculty of Medicine.

#### 2.2.1 Primary culture procedures for rat DRG neurons

**FIGURE 1:**
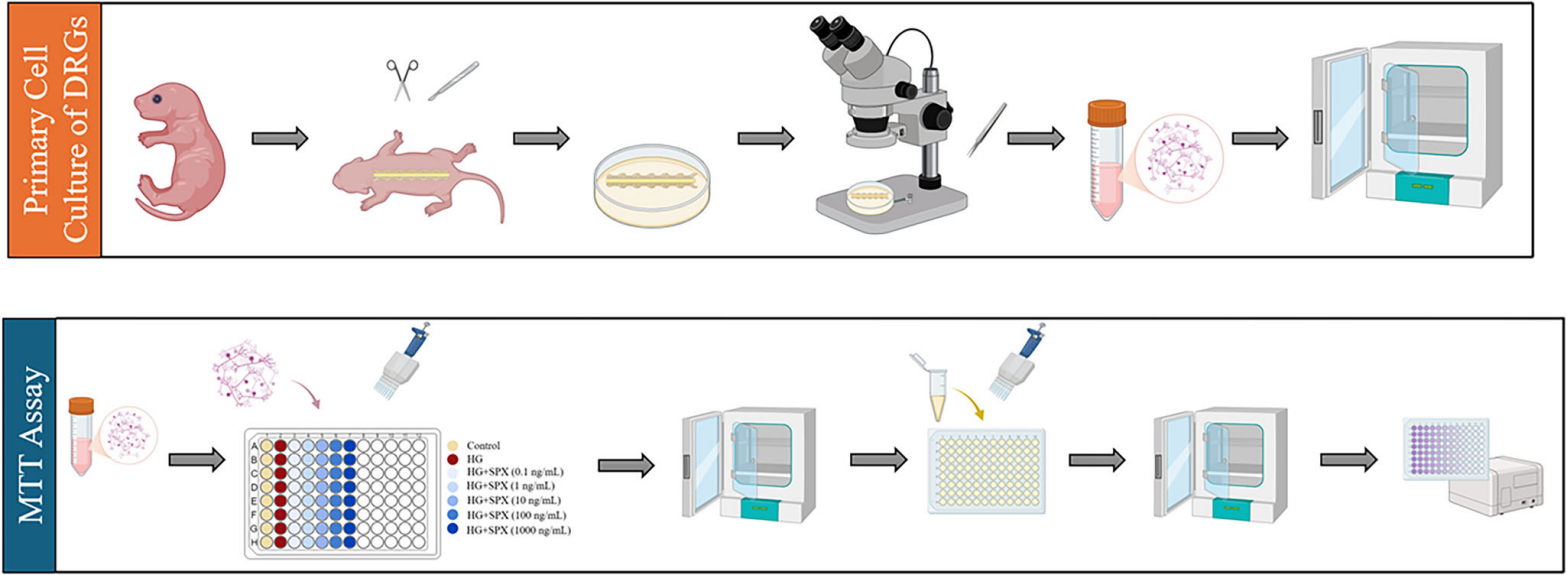
Flowchart of in vitro experimental design. HG, high glucose; SPX, spexin. Created using BioRender.com.

DRG neurons were isolated and cultured in accordance with the established protocol described by Forda & Kelly (1985). Neonatal Wistar Albino rats (postnatal days 1–2) were euthanized via decapitation to facilitate the surgical excision of the vertebral column, from which the DRGs were meticulously extracted under a dissecting microscope (Olympus, Tokyo, Japan). The isolated neuronal tissue was subjected to enzymatic digestion and mechanical dissociation to yield individual cell suspensions. These dissociated neurons were then seeded onto 96‐well plates pre‐coated with laminin‐poly‐l‐ornithine (Sigma; Steinheim, Germany). The primary cultures were maintained in a specialized medium supplemented with 2% B27 (Thermo Fisher Scientific, Waltham, MA, USA), 1% Glutamax (Thermo Fisher Scientific) and penicillin–streptomycin (5000 IU/mL; Thermo Fisher Scientific). Experimental procedures were conducted 6 h post‐seeding, with cultures kept in a humidified incubator at 37°C containing 5% CO_2_ (Ayar et al., 2014; Ozcan et al., 2010). The systematic workflow of the in vitro experimental procedures is illustrated in Figure 1.

#### 2.2.2 Viability analysis and in vitro modelling of DN in primary DRG neurons

Cellular viability was quantitatively evaluated using the 3‐(4,5‐dimethylthiazol‐2‐yl)‐2,5‐diphenyltetrazolium bromide (MTT) colorimetric assay, a widely recognized enzymatic method (Küçükbay et al., 2016). This analytical technique is predicated on the enzymatic cleavage of the tetrazolium ring within the MTT reagent (Altman, 1976). Prior to the commencement of experimental protocols, the viability of primary DRG neurons was verified via a 0.4% trypan blue exclusion test, confirming that the cell populations utilized in the study maintained a viability threshold of at least 90%. To simulate pathological conditions, an in vitro model of DN was established by exposing primary DRG cultures to high glucose (HG) concentrations (45 mmol/L) for a 24‐h incubation period (Yuksel et al., 2025).

##### 2.2.2.1 Quantification of cellular viability

Primary DRG neurons were inoculated at a density of 5×10^4^ cells per well into 96‐well microplates and subsequently subjected to treatments consisting of HG and varying concentrations of SPX (0.1, 1, 10, 100 and 1000 ng/mL) for a 24‐h duration. The experimental plates were maintained in a humidified incubator at 37°C with an atmosphere of 5% CO_2_ throughout the treatment period. Following incubation, the supernatants were removed via vacuum aspiration and 50 µL of a 0.5 mg/mL MTT solution (solubilized in sterile phosphate‐buffered saline) was added to each well using a repeater pipette. After a further 3‐h incubation phase to allow for the enzymatic conversion of the reagent, the MTT‐containing medium was aspirated and replaced with 100 µL of dimethyl sulfoxide to solubilize the resulting formazan crystals. The absorbance of the samples was subsequently quantified at a wavelength of 540 nm utilizing a spectrophotometer (Thermo Fisher Scientific) (Doğan et al., 2022; Karataş et al., 2019). To ensure statistical reliability, all in vitro cell viability assessments were evaluated with *n* = 10 independent experiments performed in triplicate. The comprehensive procedural workflow for this analysis is illustrated in Figure 1.

### 2.3 in vivo studies

#### 2.3.1 Experimental animals

The research was conducted at the Experimental Animal Production and Research Centre and the specialized laboratories of the Departments of Physiology and Histology & Embryology within Inonu University's Faculty of Medicine. While neonatal Wistar Albino rats were utilized for the in vitro primary DRG cultures to maximize neuronal yield and cell viability, BALB/c mice were specifically selected for the in vivo phase. The use of mice provided a highly validated behavioural model for STZ‐induced diabetic neuropathy while simultaneously allowing for the optimal administration of peptide quantities. A total of 40 male BALB/c strain mice (age: 6–8 weeks; weight: 30–35 g) were utilized, with the sample size determined via power analysis to ensure statistical robustness. To eliminate selection bias, animals were allocated into experimental groups using a simple random assignment computer algorithm (MedCalc 11.0 for Windows) based on their initial body weights.

Prior to the initiation of experimental protocols, the animals were housed in individual cages for a 1‐week acclimation period to adjust to the laboratory environment and to prevent social dominance‐related stress or cross‐grooming, which could significantly confound sensitive nociceptive behavioural thresholds. To mitigate the influence of confounding variables and investigator‐induced effects, behavioural assessments were performed simultaneously for all groups in a sound‐attenuated environment. All testing sessions were conducted between 09.00 and 12.00 h to ensure consistency in circadian rhythms.

#### 2.3.2 Induction and validation of the STZ‐induced in vivo DN model

Initially, normoglycaemic mice (fasting blood glucose: 70–110 mg/dL) were divided into four experimental groups: Control, DN, DN+SPX‐12.5 (12.5 µg/kg) and DN+SPX‐25 (25 µg/kg). To induce the diabetic model, streptozotocin (STZ; Sigma, Lot: WXBC0003V) was solubilized in a 0.1 M sodium citrate buffer (pH 4.5) and administered via a single intraperitoneal (i.p.) injection at a dose of 150 mg/kg to the DN and treatment groups (Oz et al., 2025; Ozcan et al., 2022; Yuksel et al., 2025). Seventy‐two hours post‐induction, fasting blood glucose (FBG) levels were quantified using a glucometer (On Call Plus, San Diego, CA, USA), with all measurements conducted between 09.00 and 10.00 h to ensure consistency. Only mice presenting FBG levels in excess of 250 mg/dL were categorized as diabetic and included in the experimental groups. Strict exclusion criteria were implemented; animals exhibiting a bodyweight reduction of more than 10%, significant hypoactivity or abnormal piloerection (stiffening of feathers/fur) prior to the treatment phase were removed from the study. Following STZ administration, a 21‐day induction period was observed to facilitate the pathophysiological development of DN (Oz et al., 2025; Yuksel et al., 2025).

#### 2.3.3 SPX administration and nociceptive behavioural assessments

Following the initial 21‐day induction period, pharmacological treatments and nociceptive behavioural evaluations were conducted over a 15‐day experimental duration, during which daily blood glucose levels were systematically monitored. SPX, solubilized in 0.9% isotonic sodium chloride, was administered via i.p. injection to the DN+SPX‐12.5 and DN+SPX‐25 cohorts at dosages of 12.5 µg/kg/day and 25 µg/kg/day, respectively. The selected SPX doses were based on previous studies (Ge et al., 2016; Lin et al., 2018). The DN group received an equivalent volume of 0.9% isotonic saline as a vehicle control, while the healthy control group remained pharmacologically untreated. Post‐injection, nociceptive thresholds were assessed across all experimental groups at predetermined temporal intervals: 0, 30, 60, 120 and 180 min. The behavioural testing battery included the hot plate and tail flick tests (single exposure per session), and the von Frey filament test, the latter performed in triplicate to derive an arithmetic mean as the definitive daily response (Oz et al., 2025; Yuksel et al., 2025).

#### 2.3.4 Assessment of thermal and mechanical nociceptive thresholds in DN

**FIGURE 2:**
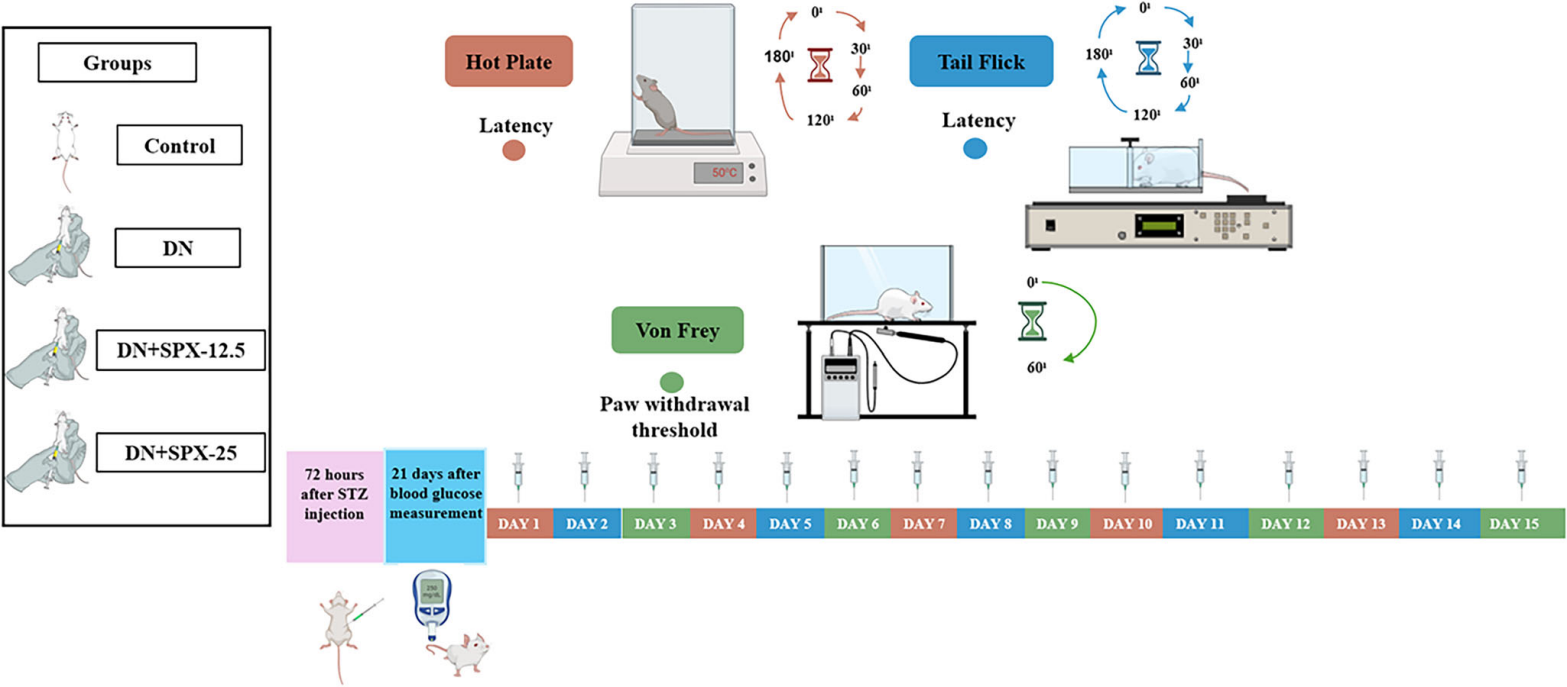
Flowchart of in vivo experimental design. DN, diabetic neuropathy; SPX: spexin; STZ, streptozotocin. Created using BioRender.com.

Behavioural evaluations of nociception were initiated 21 days following the clinical onset of diabetes to investigate the progression of DN and the therapeutic potential of SPX. To comprehensively assess thermal and mechanical hypersensitivity, three distinct behavioural paradigms were employed: the hot plate and tail flick tests for thermal nociceptive thresholds, and the von Frey filament test for the quantification of mechanical nociceptive responses. To ensure environmental acclimatization and the stabilization of behavioural responses, all subjects underwent a 1‐week adaptation period prior to the commencement of the experimental timeline. Methodological consistency was further maintained by performing five separate measurement sessions at fixed time intervals, conducted by the same investigators to mitigate inter‐observer variability. The longitudinal testing schedule was structured as follows: Day 1 was defined as the 21st day post‐diabetes induction. Thermal assessments via the hot plate test were performed on days 1, 4, 7, 10 and 13, while the tail flick test was administered on days 2, 5, 8, 11 and 14. Mechanical sensitivity was evaluated using the von Frey test on days 3, 6, 9, 12 and 15 (Oz et al., 2025). The comprehensive experimental flowchart and temporal distribution of these procedures are illustrated in Figure 2.

##### Thermal nociceptive assessment: Hot plate test

Thermal nociceptive thresholds were quantified utilizing a hot plate apparatus (Hot/Cold Plate, cat. no. 35150; Ugo Basile, Gemonio, Italy), where mice were positioned on a metallic surface maintained at a constant temperature of 50 ± 0.5°C. To ensure subject confinement and prevent escape, a transparent Plexiglas cylinder was placed over the testing area (Deuis et al., 2017). The experimental endpoint was defined as the first manifestation of thermal nociceptive behaviours, specifically hind‐paw licking, jumping, or rapid withdrawal of the claws; the elapsed time until these responses occurred was recorded as the latency period. To mitigate the risk of thermally induced tissue damage, a 60‐s cut‐off threshold was strictly implemented, and subjects failing to respond within this duration were promptly removed from the plate. Longitudinal assessments were performed at predetermined intervals – 0, 30, 60, 120 and 180 min post‐treatment – relative to a basal measurement obtained prior to any pharmacological intervention. These nociceptive evaluations were systematically recorded both in the pre‐induction phase (pre‐STZ) and following the successful establishment of the DN model.

##### Assessment of spinal nociceptive reflexes: Tail flick test

Tail‐flick latencies were quantified utilizing a radiant heat apparatus (Tail flick, cat. no. 37360; Ugo Basile, Gemonio, Italy) to evaluate spinal nociceptive reflexes. During the procedure, the distal third of the tail was exposed to a radiant thermal stimulus, with subject stability and postural standardization ensured through the use of specialized restrainers (Deuis et al., 2017). The duration until the tail withdrawal response occurred was recorded in seconds as the primary nociceptive endpoint. To mitigate the risk of thermal‐induced tissue injury, a 20‐s cut‐off threshold was strictly implemented; subjects failing to exhibit a nociceptive response within this interval were promptly removed from the apparatus. The temporal framework for these assessments included a basal measurement (pre‐injection), followed by longitudinal evaluations at 0, 30, 60, 120 and 180 min post‐intervention. To monitor the progression of sensory impairment, nociceptive data were systematically recorded both during the pre‐induction phase (prior to STZ administration) and following the successful establishment of DN.

##### Mechanical nociceptive assessment: Electronic von Frey test

Mechanical nociceptive thresholds were quantified utilizing an electronic von Frey anaesthesiometer (Electronic von Frey, cat. no. 38450; Ugo Basile, Gemonio, Italy) to evaluate tactile sensitivity. During the experimental procedure, mice were individually housed in transparent Plexiglas chambers (7 × 9 × 7 cm) positioned atop a perforated stainless steel platform (100 × 50 cm; hole diameter: 5 mm, inter‐hole distance: 2.5 mm). To ensure behavioural stabilization and environmental acclimatization, a 15‐min habituation period was observed prior to any intervention. The mechanical stimulus was applied to the plantar surface of the right hind paw; the force required to elicit a paw withdrawal reflex was quantified in G‐force (*G*). To ensure statistical robustness, measurements were conducted in triplicate for each subject, and the arithmetic mean was calculated to define the definitive nociceptive response (Oz et al., 2025; Yalcin et al., 2014). The temporal framework for these assessments included a basal measurement (pre‐injection) and a subsequent evaluation 1 h post‐injection. Furthermore, mechanical sensitivity was systematically recorded both in the pre‐induction phase (prior to STZ administration) and following the clinical establishment of DN.

#### 2.3.5 Terminal procedures and biological sample collection

Upon conclusion of the experimental period, the animals were anaesthetized with an intramuscular injection of 70 mg/kg ketamine (Richter Pharma AG, Wels, Austria) and 8 mg/kg xylazine (Alfazyne, Woerden, Netherlands). Following the induction of deep anaesthesia, the subjects were euthanized via decapitation, which was immediately followed by the systematic collection of whole blood and pancreatic tissues. Serum was isolated from the blood samples through centrifugation and subsequently cryopreserved at −80°C to ensure the stability of analytes for prospective hormonal and biochemical profiling.

The harvested pancreatic tissues were partitioned for divergent analytical modalities: a 50% representative portion was fixed in 10% neutral buffered formalin to facilitate histopathological evaluation, while the remaining 50% designated for the assessment of oxidative stress parameters was flash‐frozen and maintained at −80°C until the execution of the respective assays.

#### 2.3.6 Biochemical analyses

Quantitative biochemical assessments were executed by measuring absorbance spectra utilizing a microplate reader spectrophotometer system (BioTek Synergy HTX, Winooski, VT, USA). This instrumentation was employed to facilitate high‐throughput optical density measurements, ensuring the precision and reproducibility of the biochemical profiling performed throughout the study.

##### Evaluation of oxidative stress biomarkers

To assess oxidative stress profiles, pancreatic tissues underwent systematic homogenization and subsequent centrifugation to isolate the supernatants for downstream biochemical analysis (Çakır et al., 2019). To ensure quantitative accuracy and allow for inter‐sample comparison, total protein concentrations were determined via the Bradford method, serving as the definitive basis for data normalization (Bradford, 1976). Superoxide dismutase (SOD) activity was quantified according to the protocol established by Sun et al. (1988), utilizing a xanthine–xanthine oxidase system to generate superoxide anions and measuring the SOD‐mediated inhibition of nitroblue tetrazolium reduction at 560 nm. Catalase (CAT) activity was determined spectrophotometrically by monitoring the enzymatic decomposition rate of hydrogen peroxide (H_2_O_2_) at 240 nm (Aebi, 1984). The activities of both enzymes were normalized against protein content and expressed as U/mg protein (Cakir et al., 2015). Lipid peroxidation, indicated by malondialdehyde (MDA) levels, was evaluated via the Ohkawa et al. (1979) method; absorbance was measured at 532 nm and quantified against a standard curve in nmol/mg protein. Reduced glutathione (GSH) concentrations were assessed based on the Ellman method, whereby the reaction between GSH and 5,5′‐dithiobis‐(2‐nitrobenzoic acid) (DTNB) produced a chromogenic yellow product measured at 410 nm, with results expressed in µmol/mg protein (Gl, 1959). Furthermore, total antioxidant status (TAS; E‐BC‐K801‐M) and total oxidant status (TOS; E‐BC‐K802‐M) were quantified in serum samples utilizing specific mouse ELISA kits (Elabscience, Houston, TX, USA) in strict accordance with the manufacturer's diagnostic guidelines.

##### Quantification of serum inflammatory and anti‐inflammatory cytokines

Systemic inflammatory and anti‐inflammatory profiles were characterized by quantifying the serum concentrations of IL‐1β, IL‐6 and IL‐10. For this purpose, commercial ELISA kits specific for mouse IL‐1β (E‐EL‐M0037), IL‐6 (E‐EL‐M0044) and IL‐10 (E‐EL‐M0046) were procured from Elabscience and utilized in strict accordance with the manufacturer's diagnostic protocols. Following standardized sample preparation, serum aliquots were introduced into the ELISA plates. The procedure involved sequential incubation and stringent washing cycles, followed by the application of target‐specific biotin‐conjugated antibodies and horseradish peroxidase (HRP)–streptavidin conjugates. Chromogenic signal development was initiated via the addition of a substrate solution and subsequently neutralized with a stop solution. Absorbance was quantified using a microplate reader, and the absolute cytokine concentrations were extrapolated from the respective standard curves generated for each analyte.

#### 2.3.7 Histopathological and immunohistochemical evaluation of pancreatic tissue

Pancreatic tissue specimens harvested for histopathological examination were initially preserved in 10% neutral buffered formalin. Following standardized dehydration and clearing procedures, the tissues were embedded in paraffin wax blocks, from which serial sections of 5 µm thickness were obtained. General morphological architecture was visualized using haematoxylin–eosin (H&E) staining, while apoptotic activity was quantified through caspase‐3 immunohistochemical labelling (Çetin et al., 2016; Tekin et al., 2019). For immunohistochemical assays, sections were mounted on poly‐l‐lysine‐coated slides to ensure optimal tissue adherence. Antigen retrieval was performed by immersing the slides in citrate buffer (pH 7.6) and subjecting them to microwave irradiation for 20 min, followed by a controlled cooling period at room temperature. Endogenous peroxidase activity was neutralized via incubation in 0.3% H_2_O_2_ for 7 min. The primary labelling was executed using a polyclonal anti‐caspase‐3 antibody (Boster, Pleasanton, CA, USA; PA1302) for a duration of 2 h. This was followed by sequential 10‐min incubations with biotinylated secondary antibodies and streptavidin–peroxidase complexes. Immunoreactivity was visualized using an aminoethyl carbazole chromogen/substrate system, and sections were subsequently counterstained with Mayer's haematoxylin. Digital imaging and quantitative analysis were performed using a Leica DFC 280 light microscope integrated with the Leica Q Win image analysis system (Leica Microsystems Imaging Solutions Ltd., Cambridge, UK). The severity of pancreatic injury was systematically graded based on vascular congestion, haemorrhage, interstitial oedema, mononuclear cell infiltration, and the structural integrity of the islets of Langerhans and acinar cells, including the presence of vacuolization.

#### 2.3.8 Statistical methodologies and data visualization

In the present study, statistical computations were executed utilizing the IBM SPSS Statistics software package (Version 26.0; IBM Corp., Armonk, NY, USA) and MedCalc version 11.0 software. A semi‐quantitative scoring system was established to evaluate the staining intensity of the section planes for each preparation. The staining intensity was stratified on a scale from 0 to 3 as follows: 0 (0–25%), 1 (26–50%), 2 (51–75%) and 3 (76–100%). Automated image analysis software was not utilized for the scoring process; instead, evaluations were performed by manual microscopic inspection by a blinded investigator. Throughout the study, the sample size was strictly defined as *n* = 10 individual animals per group for in vivo experiments, and *n* = 10 independent biological experiments for in vitro assays. The normality of data distribution was rigorously assessed via the Shapiro–Wilk test. For cross‐sectional between‐group endpoints (e.g., oxidative stress markers, cytokines and histological scores), comparisons were evaluated using the non‐parametric Kruskal–Wallis H test, followed by Dunn–Bonferroni *post hoc* pairwise comparisons. To appropriately address the repeated‐measures design of the longitudinal nociceptive behavioural data, statistical analyses were conducted utilizing a linear mixed‐effects model (group × time interaction). All quantitative data are exclusively expressed as the mean ± standard deviation (SD). General statements of significance were replaced with exact *P*‐values throughout the manuscript, and statistical significance was predefined at an α‐level of 0.05. In strict accordance with data transparency standards, graphical representations were generated using GraphPad Prism software (Version 8.0; GraphPad Software, San Diego, CA, USA) as box‐and‐whisker plots with individual data points overlaid to explicitly reveal the full range and distribution of the data. A letter‐based classification system was retained in certain in vitro figures strictly as a visual guide, supplemented by exact *P*‐values.

## 3 RESULTS

### 3.1 Modulatory effects of SPX on HG‐induced DRG cell viability loss

**FIGURE 3:**
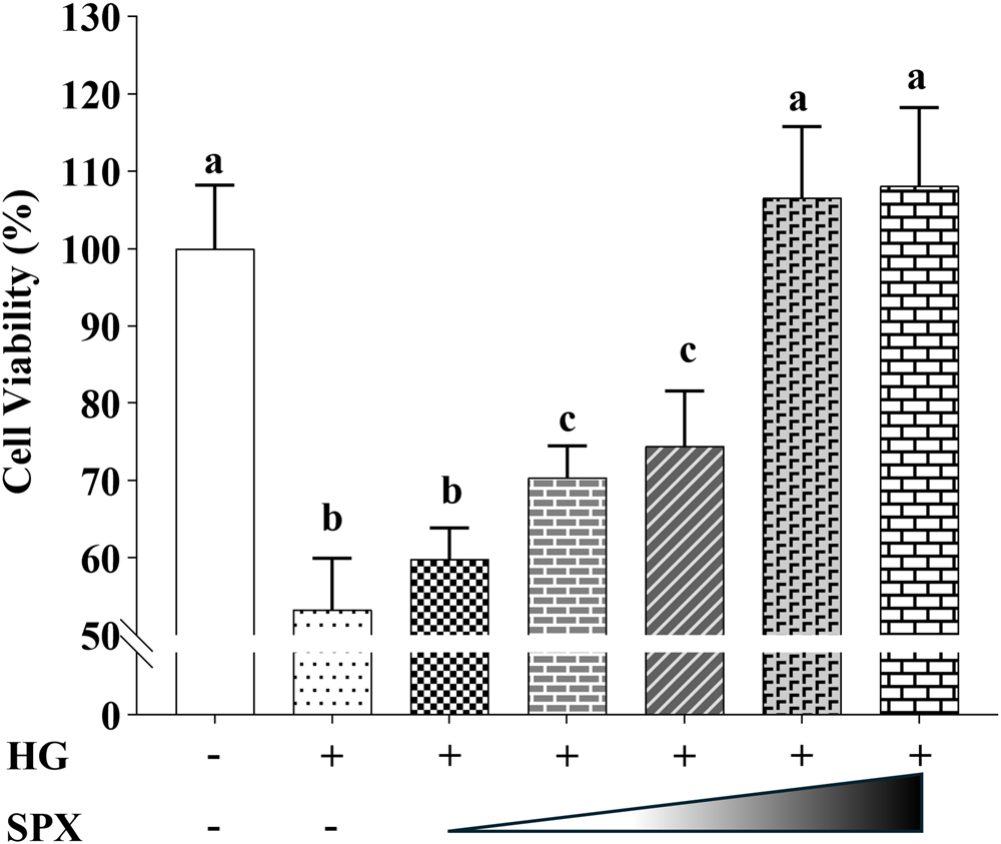
Effects of SPX on viability of HG‐exposed DRG neurons. DRG neurons were treated with HG (45 mmol/L) alone or with SPX (0.1, 1, 10, 100, 1000 ng/mL) for 24 h; an untreated Control was included. Viability assessed by MTT assay (% of Control). Data are means ± SD from *n* = 10 independent biological experiments, each performed in technical triplicate (*n* = independent experiments). Individual experiment means overlaid on bar graphs. Data were analysed using the Kruskal–Wallis test followed by the Dunn–Bonferroni *post hoc* test. HG significantly reduced viability vs. Control (*P* < 0.001). SPX ≥ 1 ng/mL significantly restored viability vs. HG (all *P* < 0.05); 0.1 ng/mL did not (*P* = 0.214). Groups sharing the same letter do not differ significantly (*P* > 0.05, Bonferroni). DRG, dorsal root ganglion; HG, high glucose; SPX, spexin.

HG exposure significantly reduced DRG cell viability compared to the Control group (*P* < 0.001). Conversely, the administration of SPX exerted a dose‐dependent restorative effect. Specifically, SPX concentrations of ≥1 ng/mL significantly restored neuronal viability compared to the HG‐exposed group; however, the 0.1 ng/mL dose did not significantly differ from the HG group (*P* = 0.214). Notably, the higher doses of SPX (100 and 1000 ng/mL) effectively normalized viability levels, with no significant differences observed when compared to the baseline values of the Control group (Figure 3).

### 3.2 Effects of SPX on glycaemic profiles in STZ‐induced diabetic models

**FIGURE 4:**
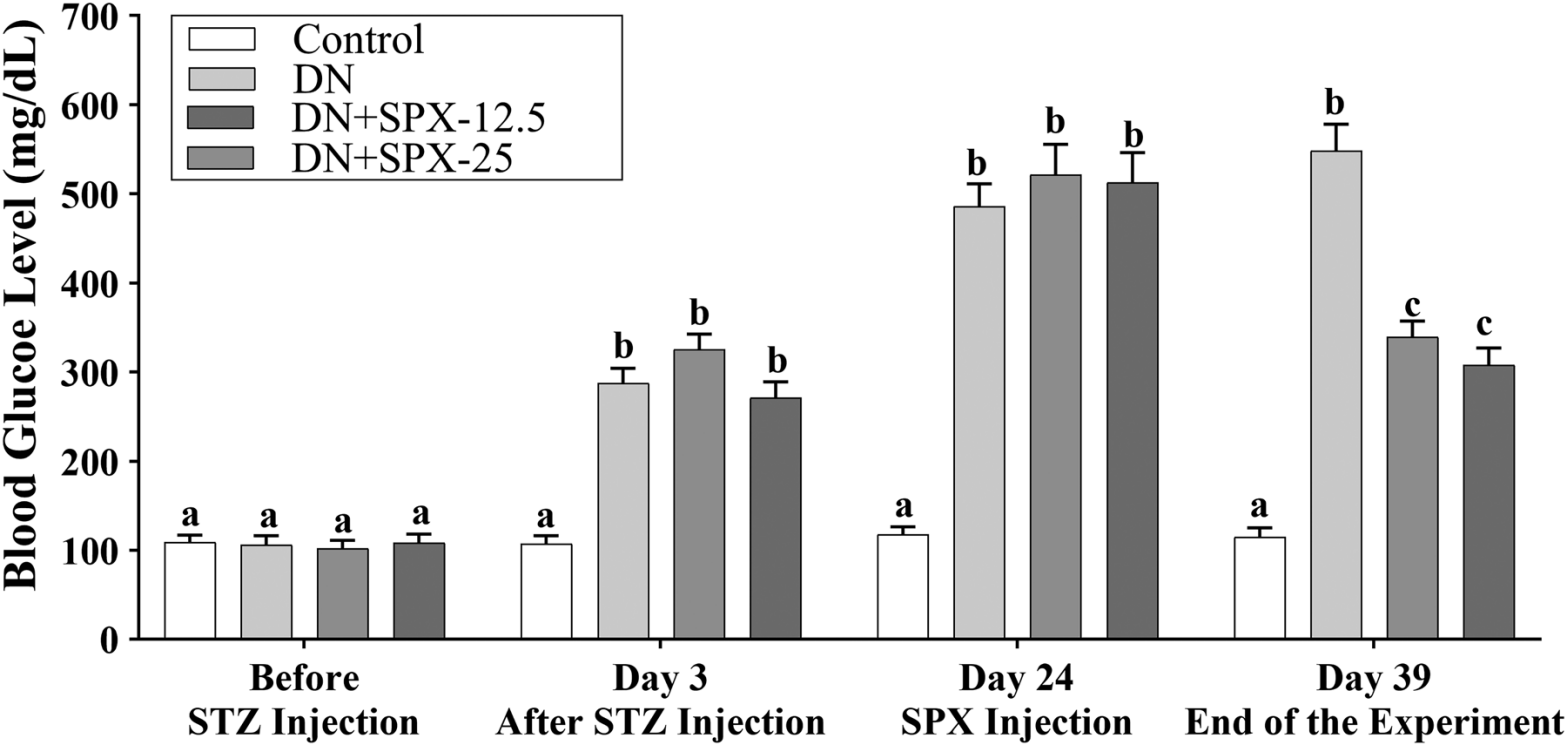
Effects of SPX on blood glucose levels in STZ‐induced diabetic mice. Blood glucose (mg/dL) was measured at baseline (before STZ), Day 3 (72 h post‐STZ), Day 24 (initiation of SPX treatment) and Day 39 (end of experiment). Data are means ± SD (*n* = 10 individual animals per group; *N* = 40); individual data points overlaid on the graphs. All groups were normoglycaemic at baseline (all *P* > 0.05). By Day 3, blood glucose was significantly elevated in DN (287.6 ± 16.7 mg/dL), DN+SPX‐12.5 (325.3 ± 17.4 mg/dL), and DN+SPX‐25 (270.7 ± 18.2 mg/dL) vs. Control (107.0 ± 9.2 mg/dL; all *P* < 0.001). At Day 39, both treatment groups showed significantly reduced blood glucose vs. DN (DN+SPX‐12.5: 339.9 ± 17.2 mg/dL, *P* < 0.001; DN+SPX‐25: 307.6 ± 19.2 mg/dL, *P* < 0.001), while remaining above Control (both *P* < 0.001). DN, diabetic neuropathy; SPX, spexin.

At baseline, all experimental groups exhibited normoglycaemic profiles. By Day 3, blood glucose levels were significantly elevated in the DN (287.6 ± 16.7 mg/dL), DN+SPX‐12.5 (325.3 ± 17.4 mg/dL) and DN+SPX‐25 (270.7 ± 18.2 mg/dL) groups compared to the Control group (107.0 ± 9.2 mg/dL; all *P* < 0.001), confirming the successful establishment of the diabetic state. Following the 15‐day therapeutic regimen (Day 39), both SPX treatment groups demonstrated a statistically significant reduction in blood glucose levels compared to the untreated DN group (DN+SPX‐12.5: 339.9 ± 17.2 mg/dL, *P* < 0.001; DN+SPX‐25: 307.6 ± 19.2 mg/dL, *P* < 0.001). Despite this marked attenuation, blood glucose concentrations in the SPX‐treated groups remained significantly higher than those of the Control group (both *P* < 0.001) (Figure 4).

### 3.3 Modulatory effects of SPX on nociceptive pain behaviour in diabetic models

**FIGURE 5:**
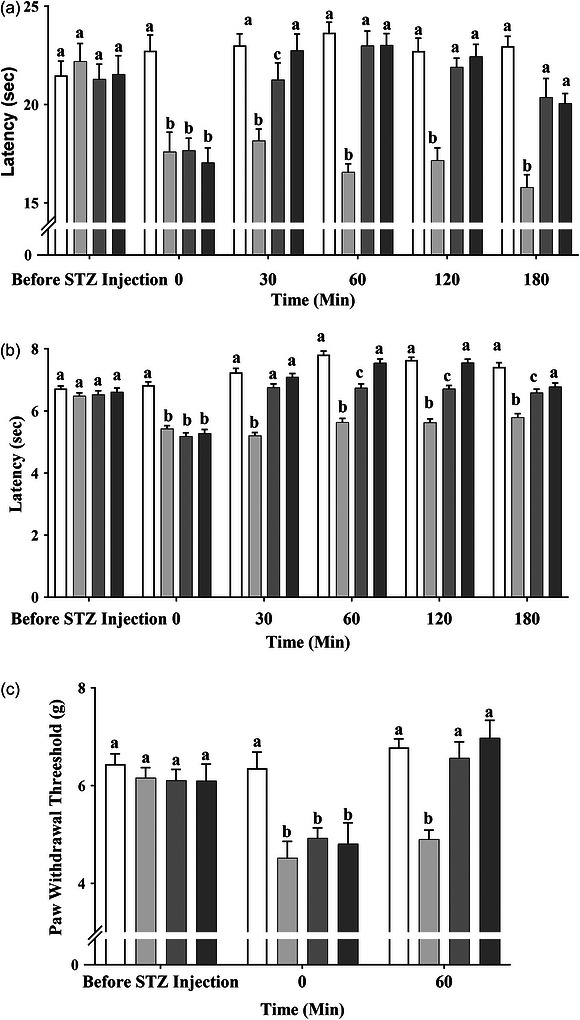
Effects of SPX on nociceptive thresholds in STZ‐induced diabetic mice. (a) Hot plate test (thermal latency, s); (b) tail flick test (spinal reflex latency, s); (c) electronic von Frey test (mechanical withdrawal threshold, g). Measurements at 0, 30, 60, 120, 180 min post‐injection on each test day. Data are means ± SD (*n* = 10 individual animals per group; *N* = 40); individual data points overlaid on bar graphs. Longitudinal data analysed using a linear mixed‐effects model (group × time interaction). Key comparisons at 60 min post‐injection – Hot plate: Control (23.64 ± 0.54 s) vs. DN (16.56 ± 0.42 s), *P* < 0.001; DN+SPX‐12.5 (23.00 ± 0.74 s) vs. DN, *P* < 0.001; DN+SPX‐25 (23.02 ± 0.59 s) vs. DN, *P* < 0.001. Tail flick: Control (7.81 ± 0.12 s) vs. DN (5.64 ± 0.12 s), *P* < 0.001; DN+SPX‐12.5 (6.75 ± 0.12 s) vs. DN, *P* < 0.001; DN+SPX‐25 (7.55 ± 0.13 s) vs. DN, *P* < 0.001. Von Frey: Control (6.78 ± 0.18 g) vs. DN (4.90 ± 0.19 g), *P* < 0.001; DN+SPX‐12.5 (6.57 ± 0.33 g) vs. DN, *P* < 0.001; DN+SPX‐25 (6.98 ± 0.36 g) vs. DN, *P* < 0.001. DN, diabetic neuropathy; SPX, spexin.

Experimental induction of diabetes (DN group) resulted in a significant attenuation of nociceptive behavioural latencies compared to the normoglycaemic Control group. At the 60‐min post‐injection interval, the DN group exhibited markedly reduced thresholds in the hot plate (16.56 ± 0.42 s vs. Control: 23.64 ± 0.54 s; *P* < 0.001), tail flick (5.64 ± 0.12 s vs. Control: 7.81 ± 0.12 s; *P* < 0.001) and von Frey tests (4.90 ± 0.19 g vs. Control: 6.78 ± 0.18 g; *P* < 0.001). Conversely, SPX treatment significantly mitigated both thermal and mechanical hypersensitivity. Thermal latencies in the hot plate test were significantly restored in the DN+SPX‐12.5 (23.00 ± 0.74 s; *P* < 0.001) and DN+SPX‐25 (23.02 ± 0.59 s; *P* < 0.001) groups compared to the untreated DN group. Similar significant improvements were observed in the tail flick latencies (DN+SPX‐12.5: 6.75 ± 0.12 s, *P* < 0.001; DN+SPX‐25: 7.55 ± 0.13 s, *P* < 0.001). Furthermore, both SPX doses significantly elevated mechanical withdrawal thresholds in the von Frey test (DN+SPX‐12.5: 6.57 ± 0.33 g, *P* < 0.001; DN+SPX‐25: 6.98 ± 0.36 g, *P* < 0.001) compared to the DN group (Figure 5).

### 3.4 Modulatory effects of SPX on pancreatic oxidative stress biomarkers

**FIGURE 6:**
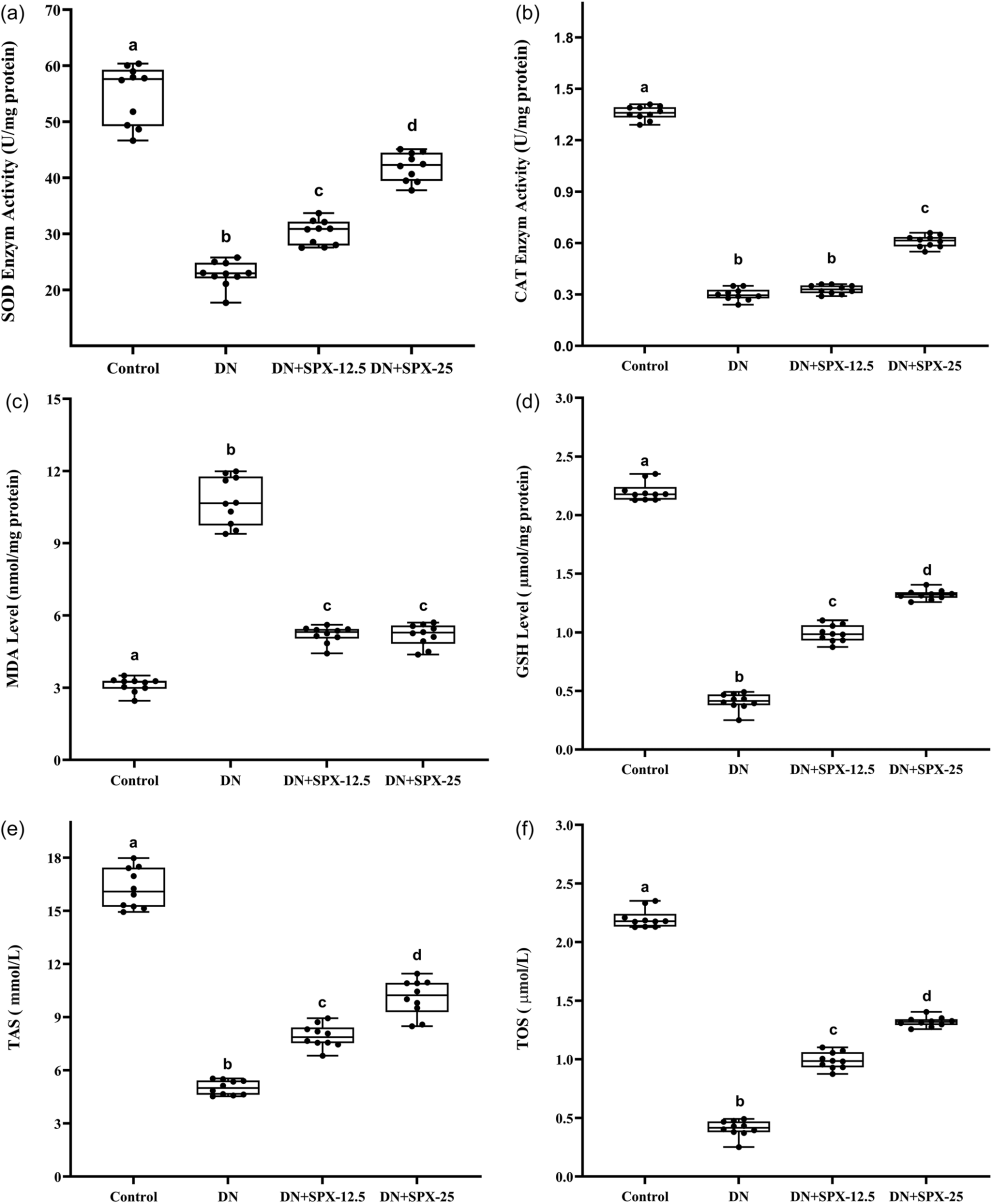
Effects of SPX on pancreatic oxidative stress biomarkers. (a) SOD, (b) CAT, (c) MDA, (d) GSH, (e) TAS, (f) TOS. Data are means ± SD; individual data points (*n* = 10 individual animals per group; *N* = 40) are overlaid on box‐and‐whisker plots (median, IQR, 1.5 × IQR whiskers). Brackets indicate significant pairwise comparisons (Kruskal–Wallis, Dunn–Bonferroni); exact *P*‐values shown above each bracket. Non‐significant pairs omitted for clarity. CAT, catalase; DN, diabetic neuropathy; GSH, glutathione; MDA, malondialdehyde; SOD, superoxide dismutase; SPX, spexin; TAS, total antioxidant status; TOS, total oxidant status.

Biochemical profiling of pancreatic tissue revealed profound, statistically significant alterations across all evaluated oxidative stress biomarkers. SOD activity exhibited significant variance among the experimental groups (*P* < 0.001). SOD levels were significantly reduced in the DN group (22.81 ± 2.28 U/mg protein) compared to the Control (54.92 ± 5.21 U/mg protein; *P* < 0.001). The administration of 25 µg/kg SPX facilitated a significant recovery (41.94 ± 2.54 U/mg protein; *P* < 0.001 vs. DN), whereas the 12.5 µg/kg dose did not yield a significant improvement (30.25 ± 2.19 U/mg protein; *P* = 0.335 vs. DN) (Figure 6a). Similarly, CAT activity exhibited significant intergroup differences (*P* < 0.001). The DN group showed markedly lower CAT activity (0.30 ± 0.03 U/mg protein) than the Control (1.36 ± 0.04 U/mg protein; *P* < 0.001). Treatment in DN+SPX‐25 group (0.61 ± 0.04 U/mg protein) significantly elevated CAT levels compared to the DN group (*P* = 0.004) and restored them to a level that did not significantly differ from the Control (*P* = 0.333). Conversely, CAT activity in the DN+SPX‐12.5 group (0.33 ± 0.03 U/mg protein) remained significantly lower than the Control (*P* < 0.001) (Figure 6b). Analysis of lipid peroxidation revealed significant alterations in MDA levels (*P* < 0.001). MDA was dramatically elevated in the DN group (10.76 ± 1.00 nmol/mg protein) relative to the Control (3.12 ± 0.30 nmol/mg protein; *P* < 0.001). Both SPX treatment groups demonstrated significantly lower MDA levels than the untreated DN group (DN+SPX‐12.5: 5.21 ± 0.35 nmol/mg protein, *P* = 0.021; DN+SPX‐25: 5.19 ± 0.46 nmol/mg protein, *P* = 0.030). Although the two treatment doses did not differ significantly from each other (*P* = 1.000), their MDA levels remained significantly above those of the Control group (*P* = 0.021 and *P* = 0.030, respectively) (Figure 6c). Endogenous antioxidant GSH levels varied significantly among the groups (*P* < 0.001). GSH was severely suppressed in the DN cohort (0.41 ± 0.07 µmol/mg protein) compared to the Control (2.20 ± 0.08 µmol/mg protein; *P* < 0.001). The DN+SPX‐25 treatment showed significant restoration of GSH against the DN group (1.32 ± 0.04 µmol/mg protein; *P* < 0.001), while the DN+SPX‐12.5 dose failed to produce a statistically significant difference from the DN group (0.99 ± 0.07 µmol/mg protein; *P* = 0.335) (Figure 6d). The overall antioxidant capacity, measured as TAS, showed significant differences among the groups (*P* < 0.001). TAS was significantly lower in the DN group (5.02 ± 0.41 nmol/mL) than in the Control (16.27 ± 1.12 nmol/mL; *P* < 0.001). The DN+SPX‐25 group exhibited a significant restoration in TAS compared to the DN group (10.11 ± 1.02 nmol/mL; *P* = 0.001), whereas the DN+SPX‐12.5 group did not significantly differ from the DN group (7.93 ± 0.64 nmol/mL; *P* = 0.280) (Figure 6e). Finally, TOS levels indicated profound systemic oxidative stress (*P* < 0.001), being sharply elevated in the DN group (9.43 ± 0.63 nmol/mL) relative to the Control (0.83 ± 0.07 nmol/mL; *P* < 0.001). Therapeutic intervention with both SPX doses resulted in TOS levels that were significantly lower than those in the DN group (DN+SPX‐12.5: 6.66 ± 0.51 nmol/mL, *P* < 0.001; DN+SPX‐25: 3.51 ± 0.22 nmol/mL, *P* < 0.001). There was no statistically significant difference in TOS reduction between the two treatment groups (*P* = 0.335) (Figure 6f).

### 3.5 Modulatory effects of SPX on systemic inflammatory and anti‐inflammatory cytokine profiles

**FIGURE 7:**
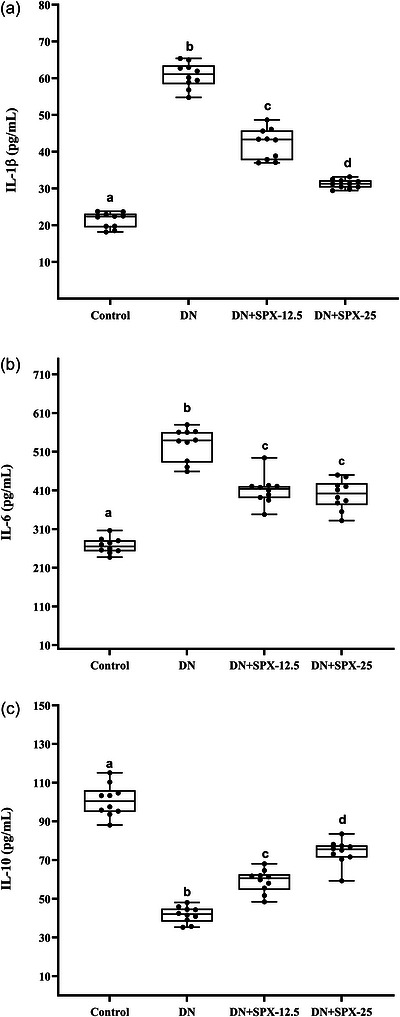
Effects of SPX on serum cytokine profiles. (a) IL‐1β, (b) IL‐6, (c) IL‐10. Data are means ± SD; individual data points (*n* = 10 individual animals per group; *N* = 40) overlaid on box‐and‐whisker plots. Brackets show significant comparisons with exact Bonferroni‐adjusted *P*‐values. Note: DN vs. DN+SPX‐12.5 for IL‐6 yielded *P* = 0.053 (not significant after Bonferroni correction; not annotated). DN, diabetic neuropathy; IL‐1β, interleukin 1 beta; IL‐6, interleukin 6; IL‐10, interleukin 10; SPX, spexin.

Biochemical analysis of systemic cytokine profiles revealed significant alterations in both pro‐inflammatory and anti‐inflammatory markers among the experimental groups. Significant variance in systemic IL‐1β levels was observed across the groups (*P* < 0.001). The pro‐inflammatory cytokine IL‐1β was markedly elevated in the DN group (60.81 ± 3.44 pg/mL) compared to the Control group (21.36 ± 2.12 pg/mL; *P* < 0.001). Both SPX treatment doses led to a significant reduction in IL‐1β levels compared to the untreated DN group (both *P* < 0.001). Notably, the IL‐1β levels in the DN+SPX‐25 group (31.23 ± 1.21 pg/mL) were robustly reduced to the extent that they did not significantly differ from the Control group (*P* = 0.335) (Figure 7a). Similarly, systemic IL‐6 levels exhibited significant intergroup differences (*P* < 0.001). The DN group (529.00 ± 42.19 pg/mL) displayed severely elevated IL‐6 levels relative to the Control group (267.00 ± 20.12 pg/mL; *P* < 0.001). Administration of 25 µg/kg SPX (399.00 ± 38.14 pg/mL) significantly attenuated this elevation compared to the DN group (*P* = 0.019). Conversely, the 12.5 µg/kg SPX dose (411.00 ± 37.21 pg/mL) did not produce a statistically significant reduction in IL‐6 levels relative to the DN group (*P* = 0.053). Despite the therapeutic effects, IL‐6 concentrations in both treatment groups remained significantly higher than those of the Control group (DN+SPX‐12.5: *P* = 0.013; DN+SPX‐25: *P* = 0.037) (Figure 7b). The anti‐inflammatory cytokine IL‐10 showed significant variation across the experimental groups (*P* < 0.001). IL‐10 concentrations were severely suppressed in the DN group (41.74 ± 4.21 pg/mL) compared to the Control group (100.71 ± 8.18 pg/mL; *P* < 0.001). Therapeutic intervention with both SPX doses effectively and significantly elevated IL‐10 levels compared to the untreated DN group (both *P* = 0.001). While the two treatment groups did not significantly differ from each other (*P* = 0.554), the DN+SPX‐25 (74.13 ± 6.39 pg/mL) treatment restored IL‐10 levels such that they did not significantly differ from the baseline Control group values (*P* = 0.256). In contrast, IL‐10 levels in the DN+SPX‐12.5 group (59.12 ± 5.92 pg/mL) remained significantly below those of the Control group (*P* = 0.001) (Figure 7c).

### 3.6 Histopathological and immunohistochemical findings in pancreatic tissue

**FIGURE 8:**
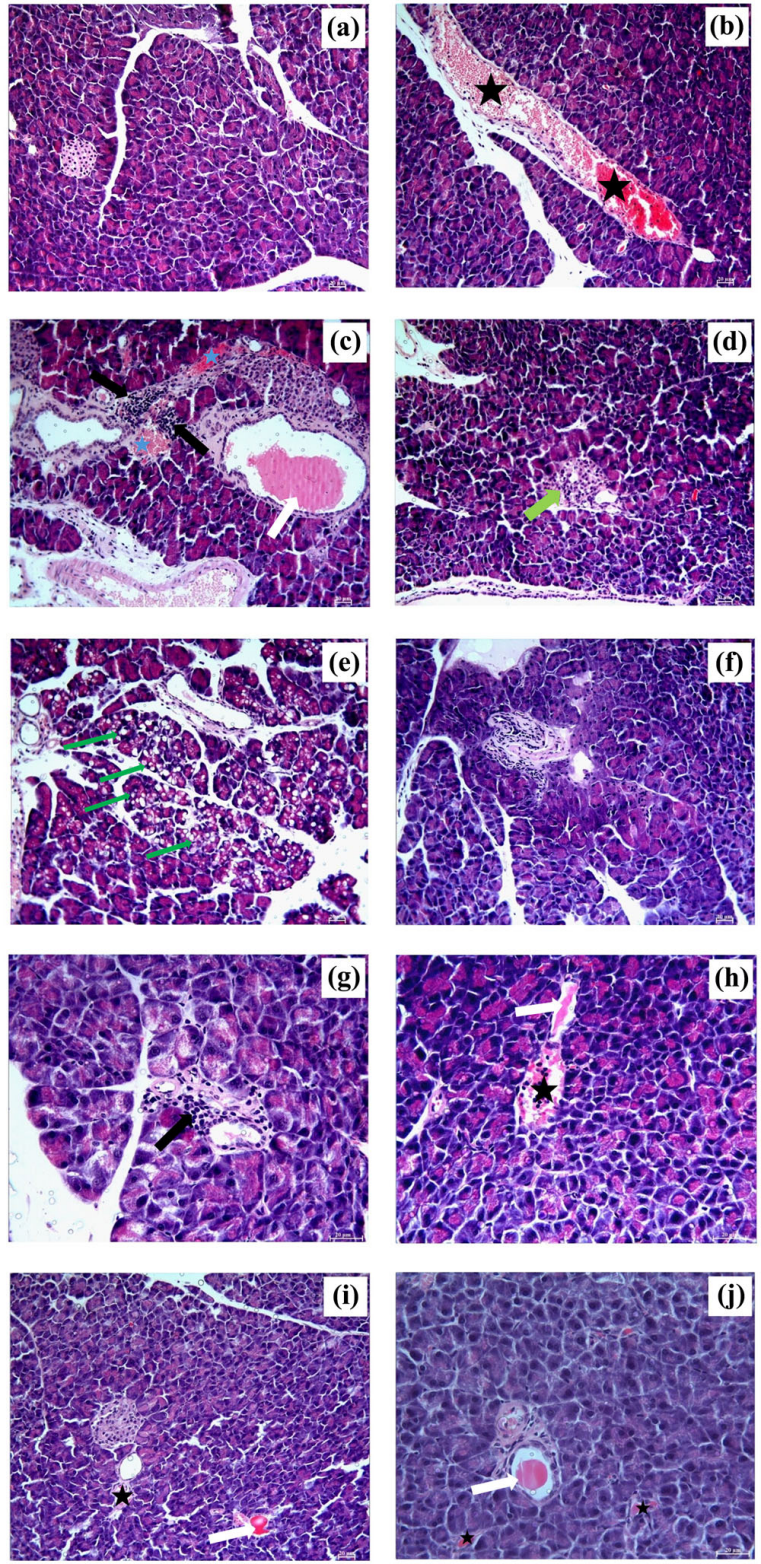
Representative H&E‐stained pancreatic tissue sections. (a) Control: normal lobular architecture, intact islets of Langerhans. (b–f) DN group: (b) vascular congestion (black star); (c) haemorrhage (blue star), oedema (white arrow), mononuclear cell infiltration (black arrows); (d) disruption of islets of Langerhans (thick green arrow) and acinar structures; (e) acinar vacuolization (thin green arrows); (f) loss of acinar integrity. (g, h) DN+SPX‐12.5: (g) minor infiltration (black arrow); (h) minor congestion (black star) and oedema (white arrows). (i, j) DN+SPX‐25: minimal congestion (black star) and oedema (white arrows); more organized morphology. Panels a, b, c, d, e, i: × 20. Panels f, g, h, j: × 40. *n* = 10 individual animals per group; representative images.

Light microscopic examination of the control group revealed a normal histological architecture of the pancreatic tissue (Figure 8a). In contrast, the DN group exhibited profound pathological alterations. These included vascular congestion (black star, Figure 8b), parenchymal haemorrhage (blue star, Figure 8c), interstitial oedema (white arrow, Figure 8c) and mononuclear cell infiltration (black arrows, Figure 8c). Additionally, structural disintegration of the Islets of Langerhans (thick green arrow) and acinar formations was observed (Figure 8d), accompanied by cytoplasmic vacuolization in acinar cells (thin green arrows, Figure 8e) and a general loss of acinar structural integrity (Figure 8f)

**FIGURE 9:**
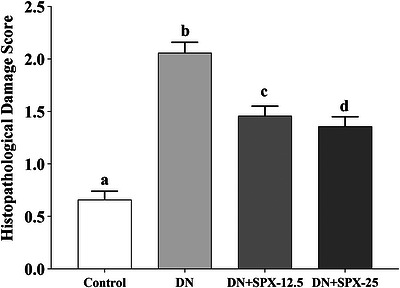
Semi‐quantitative histopathological damage scores of pancreatic tissue. Sections were graded 0–3 based on severity of vascular congestion, haemorrhage, oedema, mononuclear cell infiltration, and disruption of islets of Langerhans and acinar architecture (0: 0–25%; 1: 26–50%; 2: 51–75%; 3: 76–100% affected). Scoring by a blinded investigator; no automated image analysis. Data are means ± SD (*n* = 10 individual animals per group; *N* = 40); individual data points overlaid on box plots. Kruskal–Wallis + Dunn–Bonferroni: Control (0.66 ± 0.08) vs. DN (2.06 ± 0.10), *P* < 0.001; DN vs. DN+SPX‐12.5 (1.46 ± 0.09), *P* < 0.001; DN vs. DN+SPX‐25 (1.36 ± 0.09), *P* < 0.001; DN+SPX‐12.5 vs. DN+SPX‐25, *P* = 0.739. DN, diabetic neuropathy; SPX, spexin.

Therapeutic intervention with SPX led to a notable reduction in histopathological damage, with the most significant recovery observed in the DN+SPX‐25 group. In the DN+SPX‐12.5 group, minor instances of vascular congestion (black star, Figure 8h), limited mononuclear cell infiltration (black arrow) (Figure 8g) and slight oedema (white arrow, Figure 8h) were identified. The DN+SPX‐25 group demonstrated a more robust mitigation of tissue injury, though minimal vascular congestion (black star, Figure 8i,j) and minor oedema (white arrow, Figure 8j) persisted. Notably, the acinar structures and islets of Langerhans in this high‐dose group exhibited a more organized and regular morphology compared to the 12.5 µg/kg dose. Quantitative assessment of histopathological damage confirmed these morphological observations. The DN group exhibited significantly higher damage scores (2.06 ± 0.10) compared to the Control group (0.66 ± 0.08; *P* < 0.001). Therapeutic intervention with both SPX doses significantly mitigated this tissue damage, yielding reduced histopathological scores for both the DN+SPX‐12.5 (1.46 ± 0.09; *P* < 0.001) and DN+SPX‐25 (1.36 ± 0.09; *P* < 0.001) groups relative to the untreated DN cohort. There was no statistically significant difference in the reduction of damage scores between the two SPX treatment doses (*P* = 0.739) (Figure 9).

**FIGURE 10:**
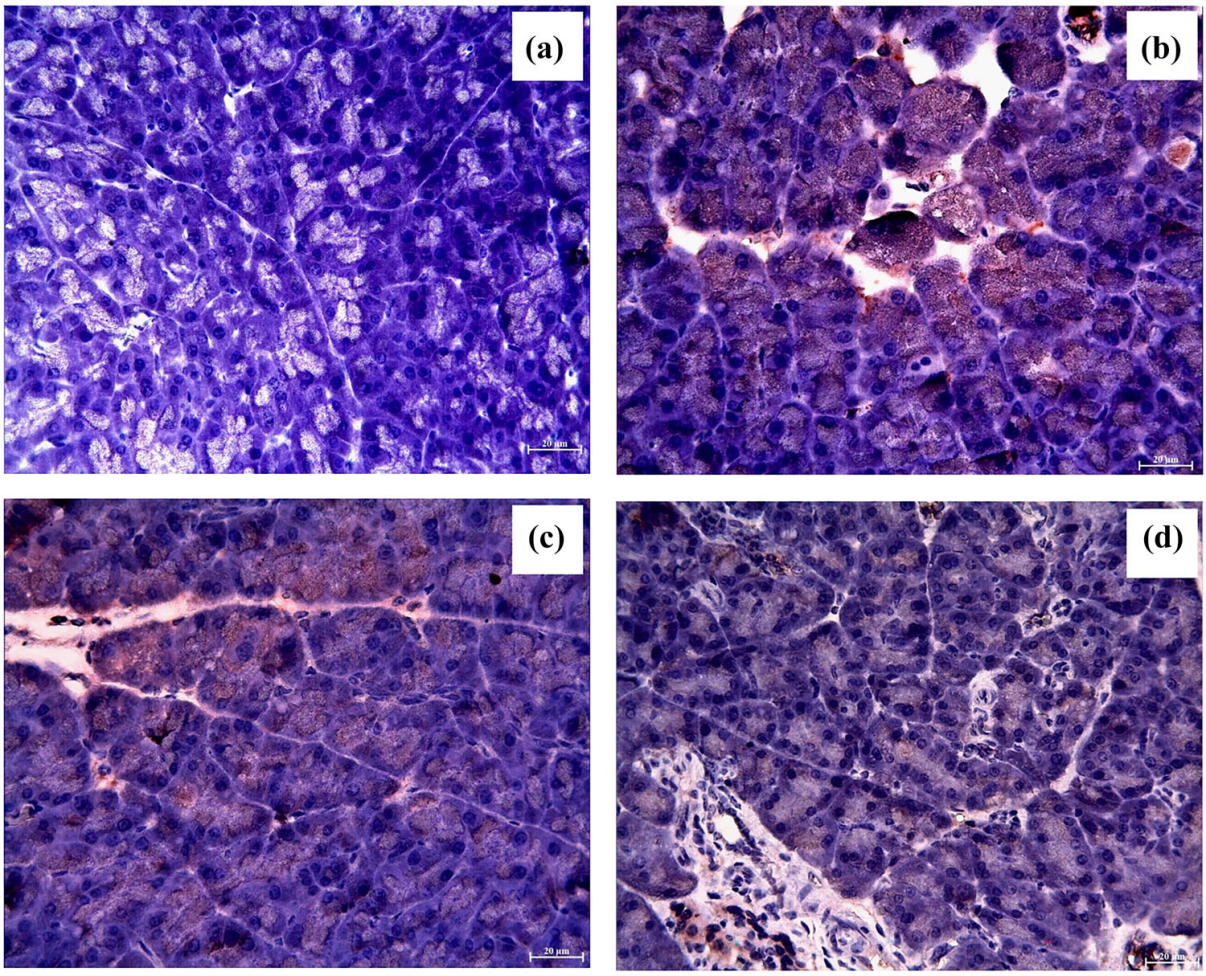
Caspase‐3 immunohistochemical staining in pancreatic sections. (a) Control, (b) DN, (c) DN+SPX‐12.5, (d) DN+SPX‐25. Caspase‐3‐positive cells (brown AEC chromogen) detected using polyclonal anti‐caspase‐3 antibody (Boster PA1302; 2 h); biotinylated secondary antibody + streptavidin–peroxidase; counterstained with Mayer's haematoxylin. DN group shows markedly elevated caspase‐3 density and staining intensity. Both SPX groups demonstrate dose‐dependent reductions; greatest decrease in DN+SPX‐25 (d). All panels: × 40. *n* = 10 individual animals per group; representative images. DN, diabetic neuropathy; SPX, spexin.

**FIGURE 11:**
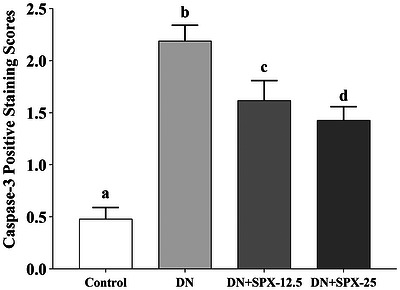
Semi‐quantitative caspase‐3 positive immunoreactivity scores (0–3; 0: 0–25%; 1: 26–50%; 2: 51–75%; 3: 76–100% positive cells). Scored by a blinded investigator. Data are means ± SD (*n* = 10 individual animals per group; *N* = 40); individual data points overlaid on box plots. Kruskal–Wallis + Dunn–Bonferroni: Control (0.48 ± 0.11) vs. DN (2.19 ± 0.15), *P* < 0.001; DN vs. DN+SPX‐12.5 (1.62 ± 0.19), *P* < 0.001; DN vs. DN+SPX‐25 (1.43 ± 0.13), *P* < 0.001; DN+SPX‐12.5 vs. DN+SPX‐25, *P* = 0.582. DN, diabetic neuropathy; SPX, spexin.

Apoptotic activity was evaluated through caspase‐3 immunoreactivity. Immunohistochemical analysis revealed a low baseline of positive cells in the Control group (Figure 10a). Conversely, the DN group was characterized by a preponderance of Caspase‐3 positive cells (Figure 10b). Following SPX administration, both the DN+SPX‐12.5 (Figure 10c) and DN+SPX‐25 (Figure 10d) groups exhibited a marked decline in the density of these apoptotic cells. Quantitative evaluation confirmed these visual observations. The DN group (2.19 ± 0.15) exhibited significantly higher caspase‐3 immunoreactivity scores compared to the Control group (0.48 ± 0.11; *P* < 0.001). Therapeutic intervention with SPX significantly attenuated apoptosis; both the DN+SPX‐12.5 (1.62 ± 0.19; *P* < 0.001) and DN+SPX‐25 (1.43 ± 0.13; *P* < 0.001) groups demonstrated significantly lower caspase‐3 scores relative to the untreated DN cohort. There was no statistically significant difference in the reduction of caspase‐3 immunoreactivity between the two SPX treatment doses (*P* = 0.582) (Figure 11).

## 4 DISCUSSION

DM is a complex metabolic disorder characterized by chronic hyperglycaemia – arising from insufficient insulin secretion, impaired insulin action, or both – which precipitates widespread metabolic dysregulation and chronic systemic complications (American Diabetes Association, 2013). Consequently, patients frequently suffer from long‐term secondary complications, most notably retinopathy, nephropathy and both peripheral and autonomic neuropathy (American Diabetes Association, 2010). Recent studies highlight that achieving strict glycaemic management is imperative not only to prevent or defer the development of DN in type 1 diabetes, but also to mitigate its progressive pathogenesis in patients with type 2 diabetes (American Diabetes Association Professional Practice Committee, 2024). SPX is a 14‐amino‐acid neuropeptide that functions as an endogenous ligand for GalR2 and GalR3 (Kim et al., 2014), with broad expression across the central nervous system and diverse peripheral tissues such as the pancreas, adrenal glands and adipose tissue (Gu et al., 2015; Porzionato et al., 2010). By activating these receptors, SPX modulates a wide array of critical physiological and metabolic processes, including feeding behaviour, reproduction, nociception, gastrointestinal motility, and cardiovascular and renal functions (Liu et al., 2013; Mirabeau et al., 2007; Rucinski et al., 2010; Toll et al., 2012; Walewski et al., 2014; Wong et al., 2013). Specifically within the endocrine pancreas, SPX suppresses glucose‐stimulated insulin secretion in cultured cells and isolated pancreatic islets, and it has been demonstrated to reduce insulin release in obese rats (Sassek et al., 2018). Furthermore, SPX is co‐localized with insulin in porcine pancreatic β‐cells, where its secretion increases shortly after glucose administration but significantly decreases during prolonged exposure (Sassek et al., 2019). Building upon these metabolic functions, our present study demonstrated that a 15‐day i.p. administration of SPX significantly reduced blood glucose levels in a mouse model of DN. This hypoglycaemic effect of prolonged SPX treatment is highly consistent with existing literature, which extensively reports that systemic SPX therapy improves glucose tolerance, alleviates insulin resistance and lowers blood glucose levels in various rodent models of diabetes and obesity.

Beyond these metabolic disturbances, the pathogenesis of DN encompasses a complex interplay of metabolic, vascular and inflammatory factors that collectively drive progressive nerve damage and neuropathic pain (Schreiber et al., 2015). Clinically, this nerve damage manifests as diabetic neuralgia, presenting as spontaneous pain characterized by a diverse spectrum of sensory abnormalities, including burning, tingling, prickling, numbness, allodynia, hyperalgesia and paresthesia (Schreiber et al., 2015). At the structural level, this condition is hallmarked by a progressive distal‐to‐proximal degeneration of the peripheral nerve fibres, which ultimately culminates in debilitating sensory and motor deficits such as chronic pain, muscle weakness and a profound loss of protective sensation (O'Brien et al., 2014). The fact that SPX operates by binding to GalR2 and GalR3 (Kim et al., 2014) makes its potential influence on pain sensitivity particularly noteworthy, especially given the established roles of these receptors in nociceptive processing (Zhang et al., 2015, 2017). In a study utilizing the tail‐flick test as an acute pain model, intracerebroventricular (i.c.v.) injection of SPX was demonstrated to exhibit antinociceptive activity (Toll et al., 2012). Furthermore, another study reported that i.c.v. administration of SPX produces an antinociceptive effect in the formalin test, which is used as a model for tonic inflammatory or visceral pain (Lv, Cui et al., 2019). Moreover, another study demonstrated that intra‐hippocampal injection of SPX, particularly when co‐administered with progesterone, significantly attenuates pain sensitivity in ovariectomized rats (Moazen et al., 2018). In the present study, we demonstrated that chronic i.p. administration of SPX for 15 days in a mouse model of DN produced significant alterations in nociceptive responses, as evaluated by the hot plate, tail‐flick and von Frey tests. These findings suggest that prolonged SPX treatment exerts a potent antinociceptive effect against DN‐induced hyperalgesia and allodynia by modulating both thermal and mechanical pain perception. Importantly, while the robust blood‐glucose‐lowering effect of SPX observed in our in vivo model undoubtedly provides a secondary systemic benefit in alleviating these neuropathic symptoms, our in vitro DRG findings confirm a direct neuroprotective mechanism. Since our in vitro findings demonstrate that SPX directly preserves DRG neuron viability under high‐glucose conditions, we propose that its antinociceptive properties are primarily mediated by direct neuroprotection, which is further enhanced by the secondary benefits of systemic glycaemic regulation.

DN also triggers a series of cellular metabolic abnormalities characterized by elevated blood glucose levels; the ensuing oxidative stress and inflammatory responses exacerbate nerve damage through the overproduction of ROS, ultimately driving neuronal apoptosis (Feldman et al., 2019). Recent studies have demonstrated that SPX ameliorates hypoxia‐induced mitochondrial dysfunction in cardiomyocytes and suppresses oxidative stress by restricting the excessive generation of ROS (Liu et al., 2020). Furthermore, SPX has been reported to exert potent antioxidant activity in models of obesity‐induced renal dysfunction (El‐Saka et al., 2023). Our findings reveal that i.p. SPX treatment exerts a robust antioxidant effect in the DN mouse model, as evidenced by the upregulation of SOD, CAT and GSH, alongside a marked reduction in MDA levels. Furthermore, the favourable shift in the oxidative balance – characterized by increased TAS and diminished TOS – provides additional evidence that SPX effectively attenuates DN‐induced oxidative stress.

Adipose tissue acts as a dynamic endocrine organ by secreting a wide array of bioactive molecules, known as adipokines, which exert pro‐inflammatory or anti‐inflammatory effects (Ouchi et al., 2011). SPX, recently identified as a novel adipokine, has been shown to ameliorate both metabolic and inflammatory profiles in obese mice; this protective effect is primarily mediated by the significant downregulation of pro‐inflammatory cytokines such as IL‐6, TNF‐α and IL‐1β, alongside the upregulation of anti‐inflammatory markers like IL‐10 (Gambaro et al., 2020). Additionally, in a rat model of adenine‐induced chronic kidney failure, SPX administration was reported to effectively modulate the systemic inflammatory response by decreasing specific pro‐inflammatory mediators, including interferon‐γ and granulocyte colony‐stimulating factor (Memi & Yazgan, 2021). In the present study, we observed that SPX administration in mice with DN led to a significant decrease in the levels of pro‐inflammatory cytokines, specifically IL‐1β and IL‐6, accompanied by an increase in the anti‐inflammatory cytokine IL‐10. Consistent with the existing literature, our data indicate that SPX is highly effective in both mitigating oxidative stress and modulating the inflammatory response in the DN model.

Although current paradigms of DN pathogenesis predominantly emphasize cellular injury resulting from hyperglycaemia‐activated cascades, the pivotal role of neuronal insulin signalling in the onset and advancement of DN is increasingly recognized (Zochodne, 2015). In this context, structural and functional damage to pancreatic β‐cells severely compromises insulin secretion, thereby driving the profound dysregulation of glucose homeostasis (Cerf, 2013). Studies have shown that SPX exerts a protective role by mitigating histopathological damage and preserving cellular integrity in kidney, liver and cardiac tissues induced by high‐fat diets and various disease (El‐Saka et al., 2023; Ge et al., 2016; Said et al., 2023). Based on our histological results, SPX treatment notably mitigated histopathological damage and reduced the expression of caspase‐3‐positive cells. Taken together, these results indicate that SPX may modulate apoptosis either by attenuating inflammatory responses driven by increased β‐cell viability and proliferation, or by limiting excessive glucose uptake into cells through improved glycaemic control.

Primary sensory neurons act as afferent pathways that relay sensory information regarding a diverse array of internal and external stimuli, including potentially damaging stimuli that result in the perception of pain, to the central nervous system (Kelestimur et al., 2021). The cell bodies of these highly compartmentalized sensory neurons are predominantly housed within the DRG or trigeminal ganglia (Krames, 2015; Mol & Roumen, 2018; Vancamp et al., 2017). Due to their exuberant metabolism and sensitivity to high glycaemia, the exposure of DRG neurons to a high‐glucose (HG) environment induces excessive ROS production within mitochondria, subsequently leading to respiratory chain dysfunction and neuronal damage (Shi et al., 2013). Consequently, DRG cells are considered to present a highly suitable in vitro cellular model for investigating peripheral nociception and the pathogenesis of neuropathy and neuronal toxicity (Kelestimur et al., 2021). Indeed, current literature consistently demonstrates that HG exposure produces oxidative damage, significantly reduces cell viability and initiates programmed cell death (apoptosis) in DRG neurons (Adam et al., 2023). Furthermore, recent investigations have revealed that various neuropeptides, such as kisspeptin and nesfatin‐1, can activate intracellular calcium signalling pathways in cultured DRG neurons, suggesting their potential to modulate somatosensory transmission and peripheral neuropathic pain (Kelestimur et al., 2021; Ozcan et al., 2016). Our findings reveal that SPX treatment effectively promotes the survival of DRG neurons subjected to HG conditions. Consequently, the results obtained herein strongly align with and substantiate current literature. By demonstrating that SPX directly preserves the viability of DRG neurons under high‐glucose conditions, our in vitro findings ensure consistency with the study's overall goals. Specifically, they confirm that the antinociceptive benefits observed in our in vivo model are not solely secondary to improved systemic glycaemic control, but are also driven by the direct neuroprotective action of SPX on primary nociceptive pathways.

In summary, the present study evaluated the therapeutic potential of SPX in a STZ‐induced DN model. Our findings reveal that SPX exerts pleiotropic effects on the underlying mechanisms driving DN pathogenesis. Notably, SPX administration significantly attenuated hyperglycaemia and improved glucose homeostasis in DN mice. Histopathological analyses demonstrated that SPX treatment mitigated pancreatic β‐cell damage, thereby preserving the structural integrity of the islets of Langerhans and notably ameliorating tissue disorganization. These morphological improvements indicate a robust cytoprotective effect of SPX on pancreatic tissue, which is crucial for maintaining glucose homeostasis. Furthermore, we provided evidence that SPX administration alleviated thermal hyperalgesia and mechanical allodynia in DN mice, thereby exerting a significant antinociceptive effect. In addition, SPX effectively modulated oxidative stress and inflammatory responses in DN mice by restoring the oxidative balance, downregulating pro‐inflammatory cytokine expression and concomitantly upregulating anti‐inflammatory cytokine levels. Taken together, these data strongly suggest that SPX represents a promising therapeutic agent for mitigating diabetic microvascular complications, oxidative stress, inflammation and apoptosis.

### 4.1 Limitations

The present study provides a comprehensive evaluation of the effects of SPX on blood glucose homeostasis, nociceptive behaviours, oxidative stress, inflammatory responses and pancreatic histopathology in an STZ‐induced DN model. However, these findings must be interpreted in light of certain limitations. First, the precise molecular mechanisms underlying the effects of SPX on glucose metabolism and neuropathic pain were not fully elucidated. Specifically, the absence of gene and protein expression profiling targeting insulin signalling cascades, glucose transporters (GLUTs), antioxidant defence systems, key inflammatory pathways (e.g., NF‐κB, MAPK) and specific upstream apoptotic markers (e.g., Bax and Bcl‐2) precludes deeper mechanistic interpretations. Second, our evaluation of oxidative stress was confined to measuring antioxidant enzyme activities and indices of lipid peroxidation. Consequently, the lack of direct quantification of cellular ROS generation represents a notable limitation. Third, the assessment of neuropathic pain relied exclusively on behavioural paradigms, without further electrophysiological or morphological analyses such as peripheral nerve conduction velocity, myelin integrity, or direct nerve tissue evaluation. Finally, DRG neurons play a pivotal role in DN pathogenesis; therefore, the failure to directly investigate the cellular and molecular cascades (e.g., oxidative stress, inflammation and cell survival pathways) modulated by SPX within these neurons constitutes a major limitation of the present work.

## AUTHOR CONTRIBUTIONS

Mehmet Refik Bahar and Suat Tekin conceptualized and designed the study. Mehmet Refik Bahar, Seval Ulku Orhan, Cigdem Tekin, Mete Ozcan and Suat Tekin performed the in vivo and in vitro experiments, and collected the data. Asli Taslidere conducted the histopathological and immunohistochemical analyses. Mete Ozcan and Suat Tekin performed the statistical analyses. Mehmet Refik Bahar wrote the first draft of the manuscript. Suat Tekin critically reviewed and edited the manuscript, acquired funding, and supervised the project. All authors have read and approved the final version of this manuscript and agree to be accountable for all aspects of the work in ensuring that questions related to the accuracy or integrity of any part of the work are appropriately investigated and resolved. All persons designated as authors qualify for authorship, and all those who qualify for authorship are listed.

## CONFLICT OF INTEREST

None declared.

## Data Availability

The data that support the findings of this study are available from the corresponding author upon reasonable request.
